# *Arabidopsis thaliana* extracts optimized for polyphenols production as potential therapeutics for the *APOE*-modulated neuroinflammation characteristic of Alzheimer’s disease *in vitro*

**DOI:** 10.1038/srep29364

**Published:** 2016-07-07

**Authors:** Shivesh Ghura, Leon Tai, Ming Zhao, Nicole Collins, Chun-Tao Che, Katherine M. Warpeha, Mary Jo LaDu

**Affiliations:** 1 Department of Anatomy and Cell Biology, College of Medicine, University of Illinois at Chicago, Chicago, IL 60612, USA; 2 Department of Medicinal Chemistry and Pharmacognosy, College of Pharmacy, University of Illinois at Chicago, Chicago, IL 60612, USA; 3 Department of Biological Sciences, University of Illinois at Chicago, Chicago, IL 60607, USA.

## Abstract

Although the cause of Alzheimer’s disease (AD) is unknown, glial-induced neuroinflammation is an early symptom. Familial AD is caused by increases in amyloid-beta (Aβ) peptide, particularly soluble oligomeric (oAβ), considered a proximal neurotoxin and neuroinflammatory stimuli. *APOE4*, a naturally occurring genotype of *APOE*, is the greatest genetic risk factor for AD; increasing risk up to 12-fold compared to *APOE3* and *APOE2.* oAβ-induced neuroinflammation is greater with *APOE4* compared to *APOE3* and *APOE2*. As sinapates and flavonoids have anti-inflammatory properties, a protocol was developed for optimizing polyphenol production in seedlings of *Arabidopsis thaliana (A. thaliana*). Three mutants (*cop1*, *prn1*, *xpf3*) were identified, and the extracts treated with liver microsomes to mimic physiological metabolism, with HPLC and MS performed on the resulting metabolites for peak identification. These extracts were used to treat primary glial cells isolated from human *APOE*-targeted-replacement (*APOE*-TR) and *APOE*-knock-out (KO) mice, with neuroinflammation induced by lipopolysaccharide (LPS) or oAβ. The dose-response data for TNFα secretion demonstrate the followed the order: *APOE*-KO > *APOE4* > *APOE3* > *APOE2*, with *xpf3* the most effective anti-neuroinflammatory across *APOE* genotypes. Thus, the plant-based approach described herein may be particularly valuable in treating the *APOE4*-induced neuroinflammatory component of AD risk.

Accumulating evidence supports the anti-oxidant and anti-inflammatory effects of polyphenols, including phenylpropanoids and its sub-classes of flavonoids, sinapate esters, and hydroxycinnamic acids, suggesting a potential role in a therapeutic approach to human inflammation-related diseases^1^,^2^, including neurodegeneration^3^,^4^,^5^. While dietary flavonoids have been shown to possess antioxidant, anti-inflammatory, and neuroprotective effects^6^,^7^, isolates of single compounds have limited success^8^,^9^. For example, both *in vitro* and *in vivo* studies of the polyphenol resveratrol indicate that half-life, bioavailability and solubility issues present major obstacles to its utility (reviewed in ref. 10). *Arabidopsis thaliana (A. thaliana)*^11^,^12^,^13^ is a useful model for the development of mutants optimized to produce polyphenols. Polyphenol-enriched extracts derived from mutants offer several advantages for the study of potential medicinal qualities of plant extracts: (1) the genome is mapped and sequenced; (2) many chemical constituents are known; (3) the mutants can be grown at a low cost, and (4) seedling growth requires only a small amount of time and space. Thus, polyphenol-enriched extracts can be easily and quickly obtained. Indeed, we recently demonstrated that specific flavonoids preferentially accumulate in the *prn1* (gene At3g59220) *A. thaliana* mutant^11^.

Alzheimer’s disease (AD) is the most commmon neurodegenerative disease of aging. Its pathological hallmarks include extracellular amyloid plaques, composed of amyloid-β (Aβ) peptide, and intracellular neurofibrillary tangles composed of abnormally phosphorylated tau protein, as well as neuron loss^14^. While amyloid plaque density may not correlate with AD-specific dementia, oligomeric Aβ (oAβ), a soluble aggregate of the peptide, has been identified as a proximal neurotoxin in human AD patients and familial AD-transgenic (FAD-Tg) mouse brains, as well as *in vitro* (reviewed in ref. 15). Importantly, *in vitro* studies demonstrate that oAβ elicits a neuroinflammatory response^16^,^17^,^18^. In AD, glial activation may be Aβ-independent, i.e. via damaged cells, or Aβ-dependent via increased soluble oAβ or amyloid plaques. Neuroinflammation due to chronically activated glia may both initiate and further exacerbate the pathogenesis of AD (for review)^19^,^20^,^21^. This creates a neuroinflammatory phenotype that is likely a driving force for early cognitive impairment^20^,^21^,^22^,^23^,^24^,^25^,^26^,^27^. Polyphenol extracts including curcumins, resveratrol and gallic acid derivatives reduce cytokine and chemokine responses in some AD models, although the specific impact on neuroinflammation is still poorly understood^28^,^29^,^30^,^31^,^32^,^33^,^34^,^35^,^36^. In addition, studies have demonstrated that dietary flavonoids preserve cognitive function during aging, and reduce the risk for AD and dementia^37^,^38^,^39^,^40^. The high consumption of polyphenolic-rich vegetables, fruit juices, and red wine has been shown to delay the onset of AD and dementia^6^,^7^,^41^,^42^. In FAD-Tg mouse models, administration of the green tea flavonoid EGCG reduced Aβ pathology with an improvement in cognition^43^,^44^, while blueberry extracts prevented deficits in cognition without altering the Aβ burden^45^. *In vitro*, flavonoids have been shown to reduce glial-mediated inflammation^46^,^47^,^48^,^49^,^50^,^51^.

The ε4 allele of the *APOE* gene is the greatest genetic risk factor for AD^52^,^53^. Risk is increased approximately 5–12-fold for carriers of one or two copies of the *APOE4* allele compared to *APOE3*, whereas *APOE2* reduces risk 2–4-fold^54^. In humans, apolipoprotein E (ApoE) has three isoforms that differ by a single amino acid substitution at residues 112 or 158: apoE2 (Cys^112,158^), apoE3 (Cys^112^Arg^158^) and apoE4 (Arg^112,158^). ApoE is the only apolipoprotein expressed within the central nervous system (CNS) and apoE-containing lipoproteins are produced primarily by astrocytes. While the impact of apoE on brain function is multifactorial, both *in vivo*^55^ and *in vitro*^18^ data demonstrate that apoE4 is associated with a greater neuroinflammatory response than apoE3^56^. While multiple lines of evidence demonstrate a heightened neuroinflammatory response with *APOE4* compared to *APOE3*, particularly in response to lipopolysaccharide (LPS; endotoxin), a ligand for the toll-like receptor 4 (TLR4) surprisingly little data address *APOE*-modulation of Aβ-induced neuroinflammation^18^. In addition, *APOE4* carriers can respond anomalously in clinical trials, sometimes negatively^57^,^58^,^59^,^60^. While epidemiological data indicate that non-steroidal anti-inflammatory drugs (NSAIDs) preferentially lower AD risk in *APOE4* carriers^61^,^62^, results from anti-inflammatory clinical trials indicate that drug efficacy will be highly context dependent (reviewed in ref. 63). For example, the conclusion from the AD Anti-inflammatory Prevention Trial (ADAPT) was that neither naproxen nor celecoxib prevented AD^64^,^65^,^66^,^67^,^68^,^69^,^70^,^71^,^72^. However, confounding factors include the short treatment duration, low AD incidence rate, and, importantly, the age of enrollment (≥70).

Thus, new therapeutic strategies targeting the neuroinflammation characteristic of AD, particularly for *APOE4* carriers, are critical. *A. thaliana* mutants may provide a novel approach for producing extracts enriched in flavonoids and polyphenols. Three such mutants were used to treat primary glial cells isolated from human *APOE*-targeted-replacement (*APOE*-TR) and *APOE*-knock-out (KO) mice, with neuroinflammation induced by LPS or oAβ. As a measure of microglial activation, tumor necrosis factor (TNF)-α, secretion followed the order: *APOE*-KO > *APOE4* > *APOE3* > *APOE2*, with *xpf3* mutant the most effective anti-neuroinflammatory across genotypes. Thus, the plant-based aproach described herein may be of economic importance to produce an effective and viable product to target neuroinflammation, one of the earliest symptoms of AD, in a form of a nutraceutical or dietary supplement.

## Results

### UV-C survival screen for *A. thaliana* mutant seedlings with increased polyphenol production

**Figure 1:**
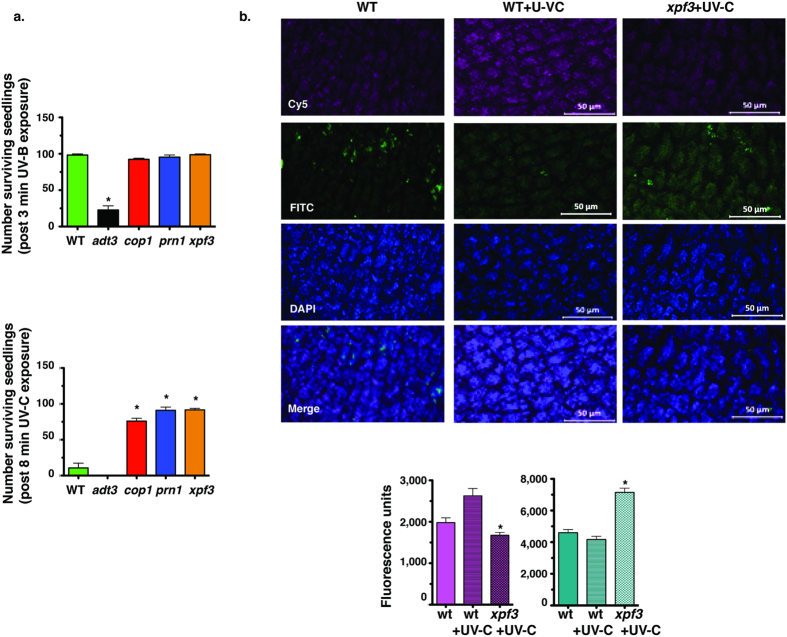
UV-C survival screen for *A. thaliana* mutant seedlings with increased polyphenol production. (**a)** 6d-old seedlings of specific *A. thaliana* mutants quantified 24 h after sub-lethal (3 min) UV-B (top) or lethal (8 min) UV-C radiation (bottom) demonstrate that mutants *cop1*, *prn1* and *xpf3* survive a dose of UV-C that is lethal to wt and the *adt3* negative control. The number of surviving seedlings for the mutants and wt, for a sub-lethal dose (top) and a lethal dose (bottom); n = 3, expressed as means ± SEM, *vs. wt. *p* < *0.05* via paired student’s t-test (two-tailed) using GraphPad Prism 5. (**b**) Post UV-C irradiation, epidermal optical slices (1 μm thick) were stained for the CellRox assay with flourescence by Cy5 indicating ROS levels (670 nm, pink,), while DAPI (358 nm; blue) and FITC (448 nm; green) capture the natural fluorescence of phenylpropanoids^11^. Representative images were captured on a deconvoluting microscopy for wt, wt + UV-C and *xpf3* + UV-C (Fig. 1B, top). Quantification of fluorescence for Cy5 (670 nm, pink, ROS) and DAPI + FITC (359–494 nm; green-blue, polyphenol levels) are represented as bar graphs (Fig. 1B, bottom). With UV treatment, the *xpf3* mutant had significantly reduced signs of oxidative stress compared to wt. The *xpf3* mutant seedlings also produced significantly more phenylpropanoids than wt. Data expressed as means ± SEM, for n = 20, *vs. wt and wt + UV-C, *p* < *0.05* via paired student’s t-test (two-tailed) using GraphPad Prism 5.

We screened 6 day (d)-old seedlings of specific mutants of *A. thaliana* and identified 3 mutants that survived UV-B irradiation comparable to wild type (wt) (Fig. 1A, top) and survived UV-C irradiation significantly better than wt and the *adt3* mutant, a mutant designed for reduced phenylalanine levels, thus the negative control (Fig. 1A, bottom). These responses are indicative of effective polyphenol accumulation or activity^11^,^73^. The mutants are: 1. Pirin1 (transcriptional co-factor and quercetinase; *prn1*); 2. constitutive photomorphogenic1 (E3 ubiquitin-protein ligase, acts as a repressor of photomorphogenesis; *cop1*); and 3. excision repair homologue 3 (homolog of the human xeroderma pigmentosum group F DNA repair; *xpf3*).

The CellRox assay was used to assess oxidative stress, an additional measure of enhanced polyphenols in the mutants, as polyphenols can be potent scavengers of reactive oxygen species (ROS). Post UV-C irradiation, epidermal optical slices (1 μm thick) were stained for the CellRox assay with flourescence by Cy5 indicating ROS levels (670 nm, pink), while DAPI (358 nm; blue) and FITC (448 nm; green) capture the natural fluorescence of phenylpropanoids^11^. Representative images were captured on a deconvoluting microscopy for wt, wt + UV-C and *xpf3* + UV-C (Fig. 1B, top). Quantification of fluorescence for Cy5 (650 nm, pink, ROS) and DAPI + FITC (359–494 nm; green-blue, polyphenol levels) are represented as bar graphs (Fig. 1B, bottom). With UV treatment, the *xpf3* mutant had significantly reduced signs of oxidative stress compared to wt. The *xpf3* mutant seedlings also produced significantly more phenylpropanoids than wt.

**Figure 2:**
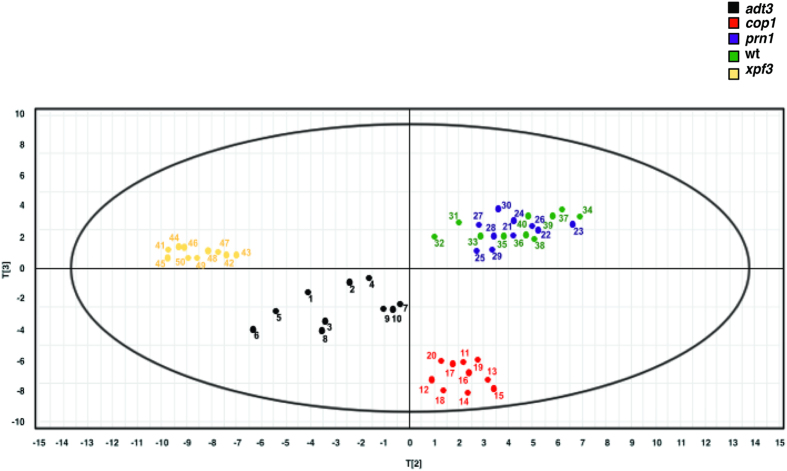
Principal component analysis (PCA) of *A. thaliana* mutants and wt indicate both batch-to-batch consistency and distinct clustering of mutants. Principal component analysis (PCA) of the mutants *xpf3*, *prn1*, *adt3*, *cop1* and wt samples, using relative content of peaks from UV spectroscopy (under UV_254 nm_, UV_280 nm_ and UV_320 nm_) as variables, demonstates that the samples formed their own cluster within a small region significantly separate from each other, with the exception of the *prn1* and wt samples. Data graphed in two dimensions using the software SIMCA-P 11.0., n = 10 batches, with each point on the graph representing a batch of the plant mutant or wt grown. The spread of the cluster corresponds to the batch to batch variability.

Using principal components analysis (PCA), independent batches of seedling extracts clustered based on mutations (Fig. 2). For PCA of the mutants *xpf3*, *prn1*, *adt3*, *cop1* and wt samples, using relative content of peaks from UV spectroscopy (under UV_254_ _nm_, UV_280 nm_ and UV_320 nm_) as variables, demonstates that the samples formed their own cluster within a small region significantly seperated from each other. The exception was the similarity of *prn1* to wt as these 2 clusters overlapped in 2-D mapping. In addition to distinguishing among the mutants, PCA also indicates the batch-to-batch consistency of a specific mutant based on the spread of the cluster.

### Identification of extractable constituents of *A. thaliana* mutant *xpf3* extract, pre- and post-treatment with liver microsomes

**Figure 3:**
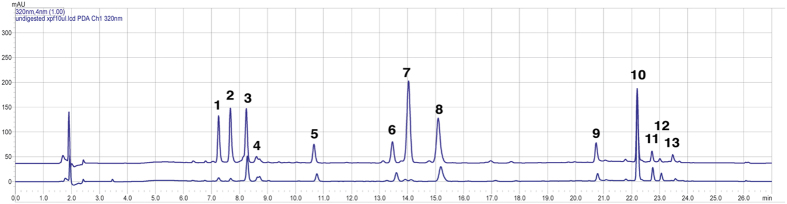
Identification of extractable constituents of *A. thaliana* mutant *xpf3* extract, pre- and post-treatment with liver microsomes. HPLC traces of the constituent chemicals from *xpf3* extracts pre and post-liver microsome (LM) treatment yielded component peaks (numbered 1–13). The upper trace is analysis for post-LM extract, the lower trace represents undigested (pre-LM) extract.

HPLC revealed 13 identifiable peaks from 10 samples of each mutant extract. A sample trace for *xpf3* extract (Fig. 3) pre- (lower) and post-(upper) treatment by murine liver microsomes (LM) used in order to mimic aspects of metabolism. LM treatment induced a quantitative increase in most of the 13 identified constituents, particularly peak 7, barely visible in the untreated extract (lower trace). The contents of the peaks were analyzed by HPLC-DAD-MS/MS (described in Methods), and structures confirmed by NMR (Table 1).

### Plant extracts dose-dependently attenuate *APOE*-modulated LPS-induced neuroinflammation *(APOE*-KO > *APOE4* > *APOE3* ≥ *APOE2)*

LM-treated mutant extracts were used to treat the primary mixed glial cultures from *APOE*-TR or *APOE*-KO mice consisting of ~95% astrocytes + 5% microglia, the approximate proportion found in both human and mouse brain. In this human apoE-relevant *in vitro* model, we measured the amount of TNFα released into the media, based on our previous work demonstrating that the levels of this cytokine are elevated significantly, likely due to the feedback loop with activated astrocytes stimulating microglia of release of TNFα. Thus, TNFα levels were a measure of the relative anti-neuroinflammatory properties of the mutant *A. thaliana* extracts. These cultures are routine in our lab^16^,^18^,^74^,^75^,^76^,^77^,^78^,^79^,^80^,^81^,^82^,^83^.

**Figure 4:**
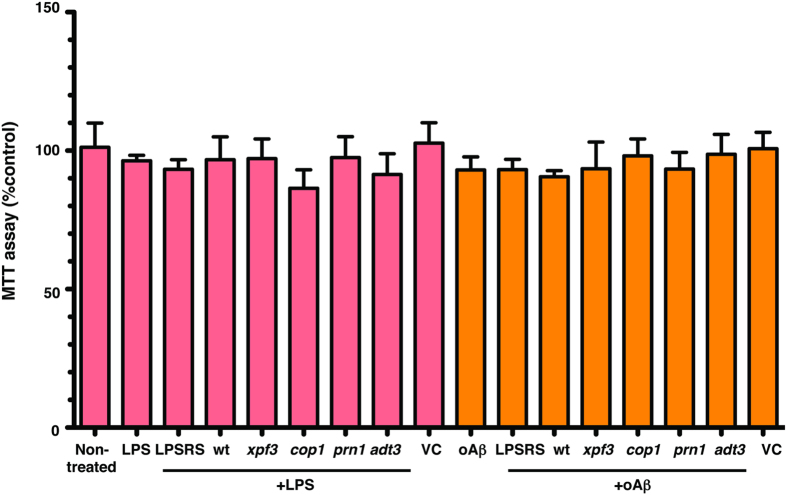
MTT assay indicates no significant cell death with LPS or oAβ treatment + /−, plant extracts at a 1:100 dose. The MTT assay, specifically an assay of mitochondrial function, was performed on mixed glial cultures from *APOE4-TR* mice, the most vulnerable genotype, treated with LM-treated plant extracts (1:100, 150 μg/ml) and an inflammatory stimuli (LPS or oAβ), demonstrates no significant cell death. Data expressed as % control for LPS or oAβ, n = 3 in duplicate for each experiment, expressed as means ± SEM.

**Figure 5:**
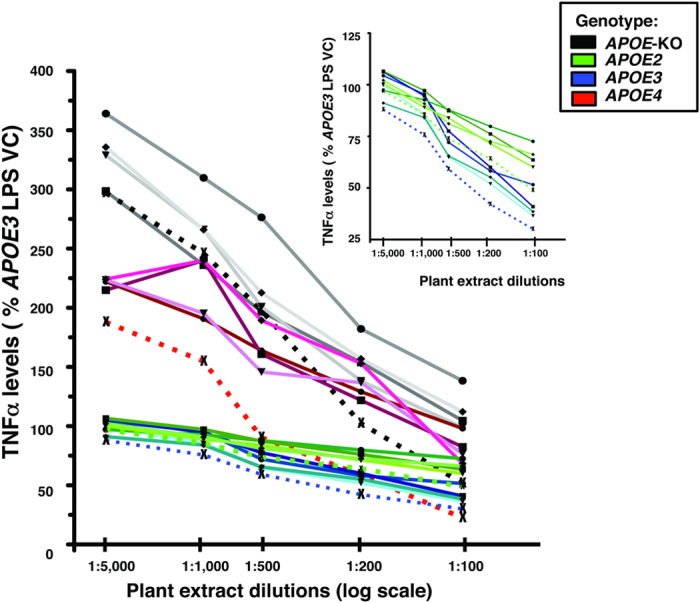
Plant extracts dose-dependently attenuate *APOE*-modulated LPS-induced neuroinflammation *(APOE*-KO > *APOE4* > *APOE3* ≥ *APOE2*). LPS-induced TNFα secretion from mixed glial cultures from *APOE*-TR or *APOE*-KO mice treated with dilutions (log scale: 1:100 to 1:5000) of plant extracts (*xpf3*, *prn1*, *cop1*, *adt3* and wt). Plant mutant *xpf3* dotted line (×), *adt3* solid line (•), wt solid line (◾), *cop1* solid line (▾), and *prn1* as solid line (♦). TNFα secretion was measured as modulated by *APOE (APOE*-KO > *APOE4* > *APOE3* ≥ *APOE2*). LPS (100 ng/ml) was added 1 h after addition of plant extracts, and TNFα levels in the media measured after 16 h by ELISA. Data are expressed as %LPS vehicle control (VC) for *APOE3*; values are an average of triplicates from a single experiment. Inset: enlargement of *APOE2* and *APOE3* cultures.

The cultures were pre-treated for 1 hour (h) with LM-digested wt, *prn1, cop1*, *xpf3* and *adt3* plant extracts (log scale dilution: 1:5000, 1:1000, 1:500, 1:200 and 1:100) prior to LPS (100 ng/ml) treatment for 16 h to elicit a neuroinflammatory response. As these are immune cells, their response to stress (LPS or oAβ) is to become activated, as evidenced by the often observed change in morphology^16^,^18^,^74^,^75^,^76^,^77^,^78^,^79^,^80^,^81^,^82^,^83^, as well as the measured secretion of TNFα. An MTT assay was preformed for cell viability, specifically mitochondral function, in the mixed glial cultures isolted from *APOE4-TR* mice, the most vulnerable genotype (Fig. 4). MTT, expressed as %control for LPS or oAβ, did not change significantly in cells treated +/− the *A. thaliana* mutant extracts at the highest dose, the 1:100 dilution. TNFα levels in the media were measured in duplicate by ELISA, and expressed as %*APOE3* LPS-vehicle control (VC) (Fig. 5). The inset is an enlarged graph that more clearly shows the separation between *APOE3* and *APOE2.* The LPS-induced inflammatory response is dose dependent and *APOE*-modulated (*APOE*-KO > *APOE4* > *APOE3* ≥ *APOE2*) with the greatest change in *APOE*-KO over the dilution range. At all dilutions, *xpf3* was effective in attenuating LPS-induced TNFα secretion and, as expected, *adt3*, was the least effective extract. Thus, the data in this figure is based on duplicates for a full dose-response experiment with LPS treated with 4 mutant + wt plant extracts in cultures from 4 *APOE* genotypes.

### *APOE*-genotype modulates LPS-induced neuroinflammation with *xpf3*-induced TNFα reduction significant with *APOE4*

**Figure 6:**
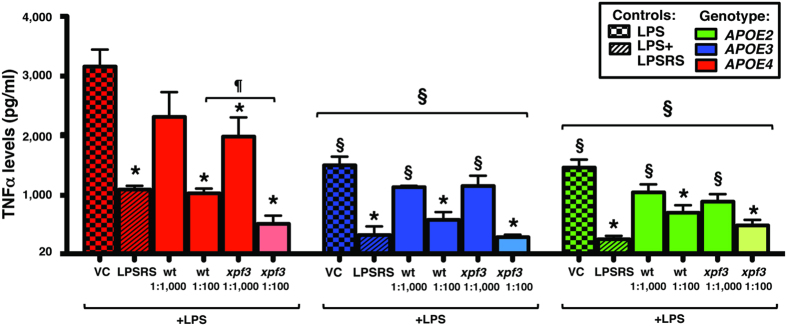
*APOE-genotype* modulates LPS-induced neuroinflammation with *xpf3-induced* TNFα reduction significant with *APOE4*. LPS (100 ng/ml)-induced TNFα secretion from mixed glial cultures from *APOE2*-, *APOE3*- or *APOE4*-TR mice treated with wt or *xpf3* plant extracts at two dilutions (1:100 and 1:1000), or LPS from *Rhodobacter sphaeroides* (LPS-RS), a natural TLR4 antagonist used as a positive control. LPS was added 1 h after addition of plant extracts, and TNFα levels in the media measured after 16 h by ELISA. Data expressed as TNFα levels (pg/ml); n = 4 in triplicate for each experiment, expressed as means ± SEM. Entire genotype effect measured via one-way ANOVA with Tukey’s post-hoc test. Differences within treatment group or within genotype measured via two-way ANOVA with Bonferroni post-hoc test. *p* < *0.05* denoted by: *vs. VC within genotype, § vs. *APOE4* and ¶vs. wt (1:100).

As described, cultures were pre-treated with LM-digested wt or *xpf3* plant extracts at two dilutions (1:100 ≈ 150 μg/ml and 1:1000 ≈ 15 μg/ml), or LPS from *Rhodobacter sphaeroides* (LPS-RS) (10 μg/ml), a natural TLR4 antagonist used as a positive control. TNFα levels in the media, were, overall, significantly higher in *APOE4* cultures compared to *APOE3* and *APOE2* cultures (Fig. 6). Within all 3 genotypes, LPS-RS, wt (1:100) and *xpf3* (1:100) were lower than the VC. Only in the *APOE4* cultures was *xpf3* (1:1000) significantly lower than VC and, importantly, *xpf3* (1:100) significanly lower than wt (1:100).

### Plant extracts dose-dependently attenuate *APOE*-modulated oAβ-induced neuroinflammation *(APOE*-KO > *APOE4* > *APOE3* ≥ *APOE2)*

**Figure 7:**
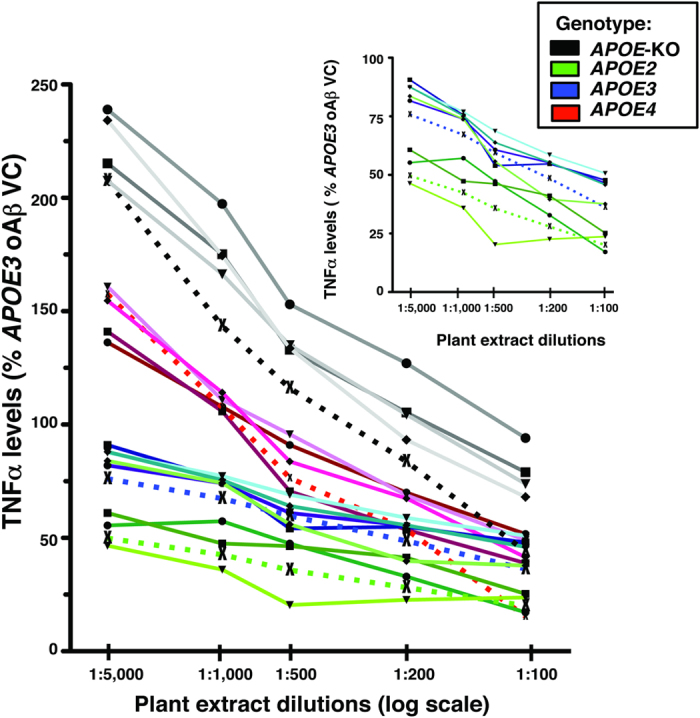
Plant extracts dose-dependently attenuate *APOE*-modulated oAβ-induced neuroinflammation *(APOE*-KO > *APOE4* > *APOE3* ≥ *APOE2*). oAβ (10 μM)-induced TNFα secretion from mixed glial cultures from *APOE*-TR or *APOE*-KO mice treated with dilutions (log scale: 1:100 to 1:5000) of plant extracts (*xpf3*, *prn1*, *cop1*, *adt3* and wt) demonstrate a dose dependent decrease in TNFα secretion modulated by *APOE (APOE*-KO > *APOE4* > *APOE3* ≥ *APOE2*). Plant mutant *xpf3* dotted line (×), *adt3* solid line (•), wt solid line (◾), *cop1* solid line (▾), and *prn1* as solid line (♦). oAβ was added 1 h after addition of plant extracts, and TNFα levels in the media measured after 16 h by ELISA. Data are expressed as % oAβ VC for *APOE3*; values are an average of triplicates from a single experiment. **Inset**: enlargement of *APOE2* and *APOE3* cultures.

As described, cultures were pre-treated with LM-digested wt, *prn1, cop1*, *xpf3* and *adt3* plant extracts (log scale dilution: 1:5000, 1:1000, 1:500, 1:200 and 1:100) prior to oAβ42 (10 μM) to induce a neuroinflammatory response. TNFα levels in the media (expressed as %*APOE3* oAβ-VC) were measured by ELISA, as shown in Fig. 7. TNFα secretion was modulated by *APOE* genotype (*APOE*-KO > *APOE4* > *APOE3* ≥ *APOE2*), with the greatest change in *APOE*-KO over the dilution range. The inset is an enlarged graph that more clearly shows the separation between *APOE3* and *APOE2.* This Figure, as well as Fig. 5 with LPS as the neuroinflammatory stimuli, illustrates the difference between the neuroinflammatory response by genotype. The response of the *APOE* genotypes to oAβ, while qualitatively similar to LPS, are aproximately ½ when expressed as TNFα leves (% *APOE3* VC) (compare Figs 5, 6, 7).

### *APOE-genotype* modulates oAβ-induced neuroinflammation with *xpf3-induced* TNFα reduction significant with *APOE4*

**Figure 8:**
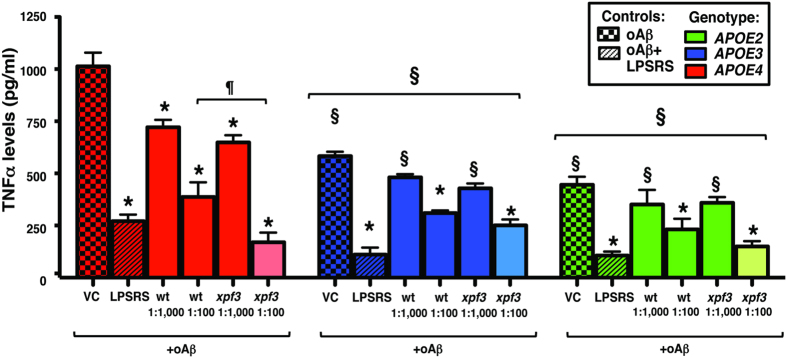
*APOE-genotype* modulates oAβ-induced neuroinflammation with *xpf3-induced* TNFα reduction significant with *APOE4*. oAβ (10 μM)-induced TNFα secretion from the mixed glial cultures from *APOE2*-, *APOE3*- or *APOE4*-TR with wt or *xpf3* plant extracts at two dilutions (1:100 and 1:1000), or LPS-RS. oAβ was added 1 h after addition of plant extracts, and TNFα levels in the media measured after 16 h by ELISA. Data expressed as TNFα levels (pg/ml); n = 4 in triplicate for each experiment, expressed as means ± SEM. Entire genotype effect measured via one-way ANOVA with Tukey’s post-hoc test. Differences within treatment group or within genotype measured via two-way ANOVA with Bonferroni post-hoc test. *p* < *0.05* denoted by: *vs. VC within genotype, §vs. *APOE4* and ¶vs. wt (1:100).

As described, cultures were pre-treated with LM-digested wt or *xpf3* plant extracts at two dilutions (1:100 ≈ 150 μg/ml and 1:1000 ≈ 15 μg/ml), or LPS-RS (10 μg/ml), and inflammation induced by oAβ (10μM). Similar to LPS, TNFα levels in the media, were, overall, significantly higher in *APOE4* cultures compared to *APOE3* and *APOE2* cultures (Fig. 8). Within all 3 genotypes, LPS-RS, wt (1:100) and *xpf3* (1:100) were lower than the VC. It is interesting that LPS-RS blocked oAβ-induced TNFα secretion in all the *APOE* cultures as LPS-RS is a TLR4 antagonist, sugesting that oAβ-induced neuroinflammation is mediated by this receptor pathway. Only in the *APOE4* cultures were wt (1:1000) and *xpf3* (1:1000) significantly lower than VC and, importantly, *xpf3* (1:100) was significanly lower than wt (1:100). As for the dose response graphs (Figs 5 and 7), the response of the *APOE* genotypes to oAβ, while qualitatively similar to LPS, are aproximately ½ when comparing TNFα levels (pg/ml) (compare Figs 6, 7, 8).

## Discussion

The studies described herein were designed to test the potential of plant-based materials for the prevention of chronic inflammation as the *A. thaliana* plant extracts were added to the cultures prior to the treatment with LPS or oAβ. Flavonoids can play critical roles in reducing negative effects of oxidative stress in plant cells, but also act as potential anti-inflammatories for human use^84^,^85^,^86^,^87^. *A. thaliana* is a genetic model with an established phenylpropanoid synthesis pathway that can be manipulated, and developed for specific chemical applications. Herein, we identified *A. thaliana* mutants, and extractable compounds that may be useful for anti-inflammatory studies in human cells. It is clear that upon intake, the polyphenols absorbed in the gut, are extensively modified^88^. The bioavailability of polyphenols differ greatly among various compounds (reviewed in ref. 89). Several *in vivo* and clinical studies have demonstrated that plant polyphenols penetrate the blood-brain barrier in substantial amounts following oral administration^90^. In the current *in vitro* study, plant extracts were treated with LM to obtain metabolites potentially active *in vivo*. LM contains enzymes like cytochrome P450s (CYP) that metabolize the plant extracts via hydroxylation and oxidative methylation^91^,^92^,^93^. Hence, the metabolic activation by LM mimic aspects of physiological ingestion and metabolism, and produce metabolites that may survive *in vivo*. This is important as some compounds lose their activity post-liver processing and metabolism, while others may assume an active form.

These data provide evidence that enhancing the anti-inflammatory properties of *A. thaliana* via polyphenols and associated molecules have potential as dietary supplements as part of a therapeutic approach to neurodegenerative disorders^1^,^2^. Furthermore, incorporating analogous mutations into consumable plants could benefit patients at a higher risk for AD. Acylation of several phenylpropanoids is typically seen in plant metabolism, dependent upon environmental cues^94^,^95^, and produces many compounds of potential importance to medicine, which may have been promoted by our growth methods in young seedlings to promote a stress-resistant (survive UV-C) chemotype. Chemicals like 1,2 di-*O*-sinapoyl-β-glucose and sinapoyl malate, known for UV-inducible responses^96^, were enhanced and accumulated in the seedling tissue under our methods, particularly in *xpf3* mutants. As well, the N,N’-di-sinapoyl spermidine, while a minority chemical in an already potent flavonoid-based extract, was elevated post LM treatment in specifically *xpf3*. Thus, the chemical analysis correlates with the function of the mutants, as mutant *xpf3* had the greatest anti-inflammatory effect, while *adt3* was the negative control with impaired polyphenol production. N,N’-di-sinapoyl spermidine is a hydroxycinnamic acid conjugated to a polyamine, referred to as hydroxycinnamic acid amide (HCAA), accumulates in *A. thaliana* seed^97^. HCAA have been implicated in oxidative stress defence in plants and utilized as a potential antioxidant, chemotherapeutic agent, and in a cardiovascular disease model^98^. This type of chemical is anti-inflammatory in human monocytes (clovamide) and mouse microglia (clovamide derivate)^99^,^100^, and neuroprotective *in vitro*^101^, suggesting a potential to reduce chronic inflammatory responses. PRN1 specifically cleaves quercetin and it is possible that while *prn1* mutants accumulate more phenylpropanoids, this increase may only be in very specific quercetin(s) according to the PCA and biological data^11^. It is currently unknown how changes in polyphenol ratios can influence inflammation, but individivual peaks are being investigated.

An important and fundamental question regarding apoE is whether the presence of apoE4 causes a loss of positive function or a gain of toxic function. For development of future therapeutics, it is critical to understand whether apoE4 represents loss of positive function or gain of toxic function. If apoE4 is a gain of toxic function, then apoE4 will induce a detrimental response compared to a lack of apoE, experimentally defined as a functional improvement in *APOE*-KO mice compared to *APOE4-TR* mice. If apoE4 is a loss of positive function, the prediction is a neutral or improved response in *APOE4*-TR mice compared to *APOE*-KO mice. Thus, a comparison between apoE4 and apoE3 for a given function is not sufficient to determine if apoE4 is worse vs. not as effective compared to apoE3. The necessary three-way comparison is *APOE*-KO vs. *APOE4* vs. *APOE3.* In this culture model, both LPS and oAβ induce neuroinflammation that is *APOE*-modulated, with TNFα secretion: *APOE*-KO > *APOE4* > *APOE3* ≥ *APOE2*, suggesting that *APOE4* confers a loss of positive function. This is consistent with previous studies of both LPS and oAβ-induced nueroinflammation *in vivo*^55^ and *in vitro* data^18^.

The effect of the apoE isoforms on activation of glial cells can vary based on the cellular composition of the culture model. *In vitro* data from astrocytes demonstrate that LPS-induced secretion of proinflammatory cytokines including TNFα, IL1β and IL6 is *APOE*-modulated, with the levels of the cytokines higher in *APOE2*, compared to *APOE3* and *APOE4*^102^. In contrast, levels of proinflammatory cytokines from LPS-induced microglial cultures are higher in *APOE4* cultures, compared to *APOE3* and *APOE2*^103^. Mixed glial cultures serve as a physiologically relevant model as it includes the individual activation of the microglia and astrocytes, as well as the paracrine-like signaling between these two major immune cells in the brain. Indeed, studies in mixed glial cultures demonstrate that LPS-induced neuroinflammation is *APOE*-modulated (reviewed in ref. 56), with levels of proinflammatory markers (TNFα, IL1β, NO, IL6, etc.) higher in *APOE4* than *APOE3*^18^,^78^,^79^,^80^,^81^. In addition, mixed glial cultures from *APOE*-KO mice showed higher LPS-induced NO production compared to glial cells from *APOE3*-TR mice^104^. Importantly, in microglia/neuron co-cultures, LPS-induced loss of synaptic protein follows the order: *APOE4* > *APOE3* > *APOE2*, recapitulated in hippocampal slice cultures^56^.

One pathway through which *APOE4* may modulate neuroinflammatory pathways is via TLR4-dependent signaling. Indeed, our published data in *APOE*/FAD-Tg mice demonstrate that microgliosis and astrogliosis are greater with *APOE4* compared to *APOE3*^105^. In addition, inflammatory mediators, as a result of TLR4 activation, are increased with *APOE4* compared to *APOE3*^18^. Complementing these *in vivo* findings, our *in vitro* data reveal that stimulation of TLR4 in mixed glial cultures with LPS or oAβ induces a similar neuroinflammatory response, as characterized by secretion of TNF-α, an effect more pronounced with *APOE4* than *APOE3*^18^. Importantly, both LPS- and oAβ-induced responses are inhibited by LPS-RS. Recent *in vitro* data also demonstrate potent TLR4 antagonism of oAβ-induced responses using the synthetic small molecule IAXO (Innaxon), developed at the University of Milano-Bicocca, and shown to inhibit innate or auto-inflammatory processes^106^,^107^,^108^.

These data can be used to further understand how to potentiate polyphenols in seedlings, and aid in the quest to develop plant-based materials suitable to prevent chronic inflammation. Furthermore, by studying the further actions of the phytochemicals, it may be possible to identify their targets and understand how they may exert their actions, as has been done for individual flavonoid chemicals *in vitro*^109^. Importantly, nutraceuticals are an inexpensive and effective option that could be potentially used to prevent neuroinflammation, hence delaying the onset of AD, specifically for *APOE4*-carriers, who are at a higher risk of both AD and non-AD *APOE4*-associated neuroinflammatory processes, including recovery from head injury, cerebral haemorrhage, stroke and cerebral artery amyloidosis.

## Conclusions

We developed a protocol for growth and extraction of high-yielding polyphenols from *A. thaliana* mutant seedlings. *An in vitro* screen using a human apoE-relevant primary glial cell model demonstrated that mutant *xpf3* (150 μg/ml) has the highest anti-inflammatory effect, as measured by TNFα lelevs in the media, compared to wt (150 μg/ml). Analysis of the ethanol extractable mutant-seedling chemicals indicated that *xpf3* possessed the highest polyphenol content per tissue mass, and had a specific polyphenol profile. In addition, the findings demonstrate that *APOE4* imparts a loss of positive function, compared to *APOE*-KO. Furthermore, oAβ induced an inflammatory response, similar to LPS, that was attenuated by LPS-RS, suggesting this response was via the TLR4 pathway. Together, this study demonstrated the use of extracts from mutant *A. thaliana* grown to maximize the polyphenol content as a model to identify plant targets that optimize the preventative strategy for AD patients, particularly for those at high risk for AD from the *APOE4* allele.

## Methods

### Chemicals

All chemicals for plant material growth and extract preparation, unless otherwise noted, were obtained from Sigma (St. Louis, MO). Aβ42 was purchased from California Peptide (now Echelon Biosciences, Salt lake city, UT; Cat. No. 641–15).

### Plant accessions

Seeds of wt Columbia (Col) *A. thaliana*, and Col mutants carrying a T-DNA insertion within the coding region *PIRIN 1 (PRN1;* At3g59220, SALK_006963)*, EXCISION REPAIR ENDONUCLEASE HOMOLOGUE 3 (XPF3*; At5g41150, (SALK_096156*), Constitutive Photomorphogenic1 (COP1*; At2g32950, SALK_022133)*, AROGENATE DEHYDRATASE_3 (ADT3*, AT2G27820; SALK_029949) were obtained from the A. thaliana Thaliana Biological Resource Center (Columbus, OH)^110^. The mutant lines are homozygous null for the reported insertions. Plants intended for seed stocks were grown as described^11^. For this study, three mutants of *A. thaliana (xpf3*, *prn1* and *cop1*) were selected based on UV-C survival (indicating polyphenol production). *adt3* was used as a negative control (produces low levels of phenylpropanoids^12^.

### Plant growth conditions for experiments

Seeds of *A. thaliana* wt or T-DNA insertion mutants were prepared, sown, and grown (seedlings are 6–7d) on 0.5 X Murashige and Skoog media 0.8% agarose phytatrays, then sealed in black Plexiglas boxes as described^11^. Cold-vernalized phytatrays were then moved to continuous darkness (Dc) at 20 °C for 6d^11^. Dc seed sets were exposed to two sublethal UV treatments of 254 nm for 3 minutes (min), 315 nm (4 min) (radiation sources described in ref. 73), with 1 min between treatments, then phytatrays returned to darkness for 12 h. Sets of live seedlings were harvested immedately after irradiations for oxidative stress studies (see oxidative stress assays), or quick frozen in liquid nitrogen for extraction. For seedling survival assays (sublethal UV-B dose and lethal UV-C dose), seedlings were treated with 254 nm as described in^11^, and survival was determined by those seedlings remaining standing and growing 24 h post-treatment, as described^73^. Those seedlings that “lodged” or fell over on the plate are characterized by major damage to the cells of the shoot and cotyledons and by 24 h and later, do not continue growing^73^.

### Oxidative stress assays

6d-old seedlings received UV treatments (described above) or control (no UV treatment). Following the treatment, live seedlings were immediately harvested into a solution of the cell-permeable fluorescent probe CellRox Deep Red^TM^, with the ROS (Life Technologies) reagent dissolved as directed into anhydrous DMSO, for 30 min incubation under rotation (50 rpm/min rotation) in darkness. Plant material was washed with PBS for 3 mins (four times) at room temperature then mounted without fixation for microscopy. The fluorescent signal from CellRox Deep Red was detected by CY5 LED (see Microscopy), and both DAPI and FITC Ex/Em data were collected for phenylpropanoid flourescence.

### Microscopy

Seedlings were viewed on a Zeiss Observer.Z1 (ApoTome 2) deconvoluting microscope. Images were collected with XCite 120 LED/Lumen Dynamics filter sets (CY5, DAPI, FITC) managed by Zen pro software (2012). Images were collected at the same exposure (control and experimental samples). At least 20 seedlings were viewed per experimental replicate. In post-processing software, fluorescence intensity was measured from at least 10 cells per cotyledon of a representative 5 imaged.

### Plant materials extraction

Aerial portions ground in liquid nitrogen were weighed, and extracted with 95% aqueous ethanol by sonication (20 ml of solvent per gram of plant material, performed twice, 2 h each treatment). After filtration, the filtrate was evaporated to remove solvent using a rotavapor under reduced vacuum, then freeze-dried. The extract was then resuspended in sterile DMSO at a constant concentration (150 mg/ml) across the plant mutant samples.

### Mixed glial cultures

Primary glial cultures (~95% astrocytes, 5% microglia) were prepared from the cerebral cortex of 1–2d old neonatal *APOE*-TR mice or *APOE*-KO mice as described^18^,^75^. Briefly, cells from *APOE2*-TR, *APOE3*-TR, *APOE4*-TR or *APOE*-KO mice were harvested and on day *in vitro* (DIV) 10–12, when the cells were confluent, they were trypsinized and plated into two 175-cm^2^ tissue culture flasks (passaged). On DIV 17–19, the secondary glial cells were plated into 24-well plates for treatment, as described below.

### Liver microsomal treatment

The plant extracts from *A. thaliana* mutants were digested using mouse LM (Gibco, Life Technologies, Grand Island, New York) to mimic mouse ingestion, as described^111^. Briefly, an ‘NADPH generating system’ was established to provide the NADPH required by the P450 enzymes in the LM. The 200 μl reaction mixture (10 μl LM, 20 μl plant extract (1:1), 1.2 μl MgCl_2_ (1 M), 6.6 μl NADP (10 mM), 6.4 μl glucose-6-phosphate (250 mM), 4.0 μl glucose-6-phosphate dehydrogenase enzyme (500 U/ml) in 152 μl HEPES buffer) was incubated at 37 °C for 30 min, followed by incubation on ice for 30 min to stop the reaction, and then centrifuged (2800 rpm, 10 min, 4 °C). The supernatant consisting of the plant extract metabolites (1:10, 15 mg/ml) potentially active *in vivo* were used for the *in vitro* treatments/screen. DMSO diluted in HEPES buffer was treated with LM, and the supernatant used as LM-vehicle control (LM-VC). The LM-VC was also used as the dilutent for the plant extract dilutions.

### HPLC and Mass Spectrometry (MS)

Ten replicates of each mutant and wt were assessed by absorbance spectrum and by HPLC for prominent peaks (before LM treatment), to evaluate reproducibility of independently grown wt and mutant samples. Data were based on the HPLC chromatogram under UV at 320 nm. Average values from 10 batches of each plant genotype were reviewed to identify peaks. Further analysis was done by MS/MS2, particularly for *xpf3*, the most effective mutant at higher dilution. *xpf3* samples before and after LM treatment were analyzed by HPLC-DAD-MS. Post-LM treatment, plant extracts were evlauated by MS. A new peak identified in *xpf3* samples was further evaluated by LC-MS/MS^2^. A Shimadzu UFLC system coupled with a LCMS-2020 detector, and an HPLC column (Agilent C_18_ column, 3 × 150 mm, 2.7 μm) were used for all HPLC-DAD-MS analyses. Acetonitrile (A) and 0.1% formic acid in water (B) were used for mobile phase and run under the gradient program as follows. The concentration of A was kept at 5% for the first min, increased to 15% for the second min, 20% for the following 13 min, 40% for 7 min and finally to increased to 100% over 5 min (then maintained a further 8 min). Flow rate, detection wavelength, and column oven temperature were set at 0.4 mL/min, 320 nm, and 27 °C, respectively. The LC-MS/MS^2^ analyses were performed on a Shimadzu LCMS-IT-TOF Mass Spectrometry.

### LPS and oAβ treatment

Secondary glial cells were seeded into 24-well tissue culture plates at a density of 1.44 × 10^6^ cells per plate. After 24 h, cells were washed with PBS to remove serum, and incubated in serum-free α-MEM media for an additional 24 h prior to treatment. The LM-digested plant extracts (*xpf3*, *prn1*, *cop1*, *adt3* and wt) or LM-VC were added to the glial cultures at the indicated dilutions, ranging from 1:100 (150 μg/ml) to 1:5000 (3 μg/ml). After 1 h, LPS (100 ng/ml, Sigma Aldrich) or oligomeric preps of Aβ42 (10 μM), prepared as described^112^, were added to the glial cultures to induce inflammation. LPS-RS (LPS from *Rhodobacter sphaeroides*, 10 μg/ml, Invivogen), a commercially available TLR4 antagonist^113^, was used as a positive control. After 16 h of incubation, conditioned media from the 24-well plates was removed, flash frozen and stored at −80 °C. TNFα levels in the media were measured using a mouse TNFα ELISA (Invitrogen), following the manufacturer’s protocol. The TNFα levels are expressed as %LPS VC, or oAβ VC of each *APOE* genotype relative to the *APOE3* VC.

### MTT Assay

The MTT assay, a measure of cell viability that specifically measures mtichondrial function, was performed on mixed glial cultures isolated from *APOE4*-TR mice, the most vulnerable APOE genotype. Cultures were treated with LM-digested plant extracts (1:100, 150 μg/ml) and an inflammatory stimuli (LPS or oAβ). Manufacturers protocol was followed for the assay (Catalog No CT02, EMD Millipore, Massachusetts). Briefly, the conditioned media from mixed glial cultures was removed and 0.2 ml of the MTT solution (10% MTT in serum free media) was added to the cells. After 4 h incubation at 37 C, the media was gently removed and 0.5 ml isopropanol was added to each well. The absorbance was measured at 570 nm. The absorbance values are expressed as % of non-treated cells (% control) for LPS or oAβ.

### Statistical analysis

Statistical significance was determined using GraphPad Prism version 5 for Macintosh. For microscopy data of Fig. 1, Graphpad was used for fluorescence measurement values, using two-tailed t-test with Welch’s correction. For Fig. 4, the MTT assay was performed in duplicate, n = 3 independently replicated experiments. For Figs 5 and 7, the experiments were performed in duplicate to obtain dose curves for LPS- and oAβ-induced inflammation using the five plant extracts (*xpf3*, *prn1*, *cop1*, *adt3* and wt), at five dilutions (log scale: 1:5000, 1:1000, 1:500, 1:200 and 1:100), for each *APOE* genotype (*APOE2*, *APOE3*, *APOE4* and *APOE*-KO). For Figs 6 and 8, data are presented as mean ± SEM; experiments were performed in triplicate, n = 3–4 independently replicated experiments (described in Fig. Legends). Entire genotype effect measured via one-way ANOVA with Tukey’s post-hoc test. Differences within treatment group or within genotype measured via two-way ANOVA with Bonferroni post-hoc test. *p* < *0.05* denoted by: *vs. VC within genotype, §vs. *APOE4* and ¶vs. wt (1:100).

## Additional Information

**How to cite this article**: Ghura, S. *et al*. *Arabidopsis thaliana* extracts optimized for polyphenols production as potential therapeutics for the *APOE*-modulated neuroinflammation characteristic of Alzheimer’s disease *in vitro*. *Sci. Rep.* **6**, 29364; doi: 10.1038/srep29364 (2016).

## Figures and Tables

**Table 1:** Peak identification by HPLC-DAD-MS/MS analysis.

Peak	Identification	M.F./M.W.			MS (+) (m/z)		MS (−) (m/z)	
			RT (min)	UVmax (nm)	MS1	MS2	MS1	MS2
1	Isomer of 1-O-sinapoyl-*β*-D-glucose	C_17_H_22_O_10_/386	7.187	234, 323	369 [M-H_2_O+H]^+^	–	385 [M-H]^−^	–
2	Isomer of 1-O-sinapoyl-*β*-D-glucose	C_17_H_22_O_10_/386	7.588	224, 327	369 [M-H_2_O+H]^+^	–	385 [M-H]^−^	–
3^a^	1-O-Sinapoyl-*β*-glucose	C_17_H_22_O_10_/386	8.129	237, 332	369 [M-H_2_OH_2_O+H]^+^	–	385 [M-H]^−^	–
4	Sinapoyl choline	C_16_H_24_NO_5_/310	8.473	236, 327	310 [M]^+^	251, 207	–	–
5^a^	Quercetin 3-O-*β*-glucoside-7-O-*α*-rhamnoside	C_27_H_30_O_16_/610	10.322	255, 353	611 [M+H]^+^	303	609 [M-H]^−^	–
6	Quercetin 3,7-di-O-*α*-rhamnoside	C_27_H_30_O_15_/594	12.985	255, 347	595 [M+H]^+^	303	593 [M-H]^−^	–
7^b^	N,N′-Di-sinapoyl spermidine	C_29_H_39_N_3_O_8_/557	13.763	241,3 23	558 [M+H]^+^	352, 264	–	–
8	Sinapoyl malate	C_15_H_16_O_9_/340	14.739	238, 329	–	–	339 [M-H]^−^, 679 [2M-H]	223
9	Isomer of 1,2-di-O-sinapoyl-*β*-glucose	C_28_H_32_O_14_/592	20.494	239, 327	369 [M-C_11_H_11_O_4_-H_2_O+H]^+^	207, 175	591 [M-H]^−^	349, 367
10^a^	1,2-Di-O-sinapoyl-*β*-glucose	C_28_H_32_O_14_/592	22.022	238, 329	369 [M-C_11_H_11_O_4_-H_2_O+H]^+^	351, 207, 175	591 [M-H]^−^	349, 367
11	Isomer of 1,2-di-O-sinapoyl-*β*-glucose	C_28_H_32_O_14_/592	22.563	239, 328	369 [M-C_11_H_11_O_4_-H_2_O+H]^+^	351, 207, 175	591 [M-H]^−^	349, 367
12	Isomer of 1,2-di-O-sinapoyl-*β*-glucose	C_28_H_32_O_14_/592	22.854	240, 327	369 [M-C_11_H_11_O_4_-H_2_O+H]^+^	351, 207, 175	591 [M-H]^−^	349, 367
13	Isomer of 1,2-di-O-sinapoyl-*β*-glucose	C_28_H_32_O_14_/592	23.334	241, 327	369 [M-C_11_H_11_O_4_-H_2_O+H]^+^	351, 207, 175	591 [M-H]^−^	349, 367

^a^The structures were further confirmed by ^1^H NMR data.

^b^N,N′-Di-sinapoyl spermidine or one of its isomers, different in the positions of OCH_3_ and OH groups.
